# Drug-associated serotonin syndrome in elderly patients: a comprehensive disproportionality analysis based on the FAERS database

**DOI:** 10.3389/fphar.2025.1700469

**Published:** 2025-10-21

**Authors:** Xiaozhi Li, Xiaofen Wen, Hongbo Fu, Xiaobing Hong, Danxia Lin, Xiaoyan Li, Zhikun Liang, Mengna Wang, Xihui Yu, Yanqiong Zhou, Xiajiong Luo

**Affiliations:** ^1^ Department of Medical Oncology, Cancer Hospital of Shantou University Medical College, Shantou, China; ^2^ Department of Pharmacy, The Second Affiliated Hospital of Shantou University Medical College, Shantou, China; ^3^ Department of Pharmacy, The Sixth Affiliated Hospital of Sun Yat-Sen University, Guangzhou, China; ^4^ Department of Pharmacy, Guizhou Hospital of The First Affiliated Hospital, Sun Yat-sen University, Guiyang, China; ^5^ Department of Pharmacy, The Affiliated Hospital of Guizhou Medical University, Guiyang, China

**Keywords:** elderly Patients, drug-Associated, serotonin syndrome, pharmacovigilance, FARES, ADEs

## Abstract

**Background:**

This study aims to identify drugs associated with serotonin syndrome in elderly patients by utilizing the FDA Adverse Event Reporting System (FAERS), to provide evidence-based references for safe clinical medication practices.

**Methods:**

This study analyzed data extracted from the FAERS covering the period from Q1 2004 to Q1 2025, with the objective of identifying drugs associated with serotonin syndrome in elderly patients. Disproportionality analysis was utilized to detect potential drug-associated signals, and sensitivity analyses were performed to evaluate the stability and strength of serotonin syndrome signals associated with these drugs. Time-to-onset (TTO) analysis was conducted to investigate factors influencing the clinical presentation of serotonin syndrome.

**Results:**

Disproportionality analysis identified 68 drugs associated with serotonin syndrome in elderly patients. Among the drugs with positive signals, the most frequently reported category associated with serotonin syndrome in elderly patients was nervous system drugs, followed by antiinfectives for systemic use, alimentary tract and metabolism drugs, musculo-skeletal system drugs, dermatologicals, and respiratory system drugs. Sensitivity analyses confirmed that most positive signals remained robust. TTO analysis revealed that drug-associated serotonin syndrome onset occurred earlier in elderly female patients.

**Conclusion:**

Drug-associated serotonin syndrome risk is elevated among elderly patients. Prompt identification and discontinuation of the causative drugs are crucial for the effective management of serotonin syndrome. In clinical practice, the risk of drug-associated serotonin syndrome should be taken into account to optimize pharmacotherapy.

## 1 Introduction

Serotonin syndrome is a toxic syndrome characterized by excessive 5-hydroxytryptaminergic (5-HT) activity. It primarily stems from elevated synaptic concentrations of 5-HT, with selective involvement of the brainstem and spinal cord (Dunkley et al., 2003). It may result in prolonged hospitalizations and even mortality. Clinically, the condition is characterized by a classic symptom triad: altered mental status, autonomic hyperactivity, and neuromuscular dysfunction. Common manifestations include agitation, myoclonus, hyperreflexia, diaphoresis, tremors, and fever (Boyer and Shannon, 2005; Birmes et al., 2003; Mason et al., 2000; Bodner et al., 1995); in severe cases, it may be life-threatening (Prakash et al., 2021a). Notably, no correlation exists between serum 5-HT levels and clinical manifestations, and there is currently no validated laboratory test to confirm the diagnosis. (Mason et al., 2000); diagnosis is primarily based on a comprehensive assessment of patients' clinical symptomatology and medication exposure history. Therefore, the comprehensive identification of drugs associated with serotonin syndrome is of critical clinical importance (Wang et al., 2016; Tormoehlen and Rusyniak, 2018).

The true incidence of serotonin syndrome remains unclear; however, it is generally regarded as low (Moss and Hendrickson, 2019; Duignan et al., 2020; Nguyen et al., 2017). Studies have reported that the incidence of serotonin syndrome among patients receiving serotonergic drugs ranges from 0.07% to 0.19% (Werneke et al., 2020). In recent years, with the widespread use of serotonergic drugs, the risk of serotonin syndrome has increased (Boyer and Shannon, 2005; Francescangeli et al., 2019; Mason et al., 2000; Mojtabai and Olfson, 2014; Scotton et al., 2019; Sternbach, 1991), particularly among elderly patients with polypharmacy (Erken et al., 2022; Poeschla et al., 2011). Research indicates that the prescription rates of potent serotonergic opioids, such as tramadol, have been increasing annually in the elderly population, and their use alone or in combination with other serotonergic drugs has emerged as a significant trigger for serotonin syndrome in this population (Mullins et al., 2022; Ruiz De Villa et al., 2021; Hardy and Panikkar, 2020; Eksi et al., 2019; Shahani, 2012; Peacock and Wright, 2011).

However, the diagnosis of serotonin syndrome in elderly patients is more challenging than that in other age groups. A study enrolling 309 intensive care unit patients showed that 8% of the patients met the Hunter Serotonin Toxicity Criteria for serotonin syndrome, with 58.3% aged ≥55 years; yet, none of these cases had been correctly diagnosed prior to evaluation by the researchers (Prakash et al., 2021b). Misdiagnosis of serotonin syndrome or failure to recognize underlying drug reactions may lead to the occurrence or exacerbation of serotonin syndrome (Prakash et al., 2021b).

Despite the critical importance of identifying drug-associated serotonin syndrome in elderly patients, current clinical evidence remains markedly insufficient. The FDA Adverse Event Reporting System (FAERS) stands as one of the world’s leading adverse event reporting databases, which provides postmarketing drug safety information to healthcare professionals and the public through the voluntary MedWatch adverse event reporting program (Sakaeda et al., 2013). Recent analyses of adverse event data from the FAERS have confirmed that selective serotonin reuptake inhibitors (SSRIs) and monoamine oxidase inhibitors (MAOIs) represent the primary drug classes implicated in the induction of serotonin syndrome. Notably, an appreciably higher pharmacovigilance signal for this condition has been observed in elderly individuals (Elli et al., 2024). However, this study neither conducted a dedicated in-depth analysis targeting the elderly, a high-risk group, nor explored the onset time characteristics of serotonin syndrome. This study aims to identify drugs associated with serotonin syndrome in elderly patients by utilizing the FAERS, identify potential drug-associated risk signals for increased serotonin syndrome risk in elderly populations, determine the risk magnitudes of such associations, and provide evidence for clinical medication decision-making and serotonin syndrome event reduction, ultimately enhancing medication safety in elderly patients.

## 2 Methods

### 2.1 Data source

**FIGURE 1:**
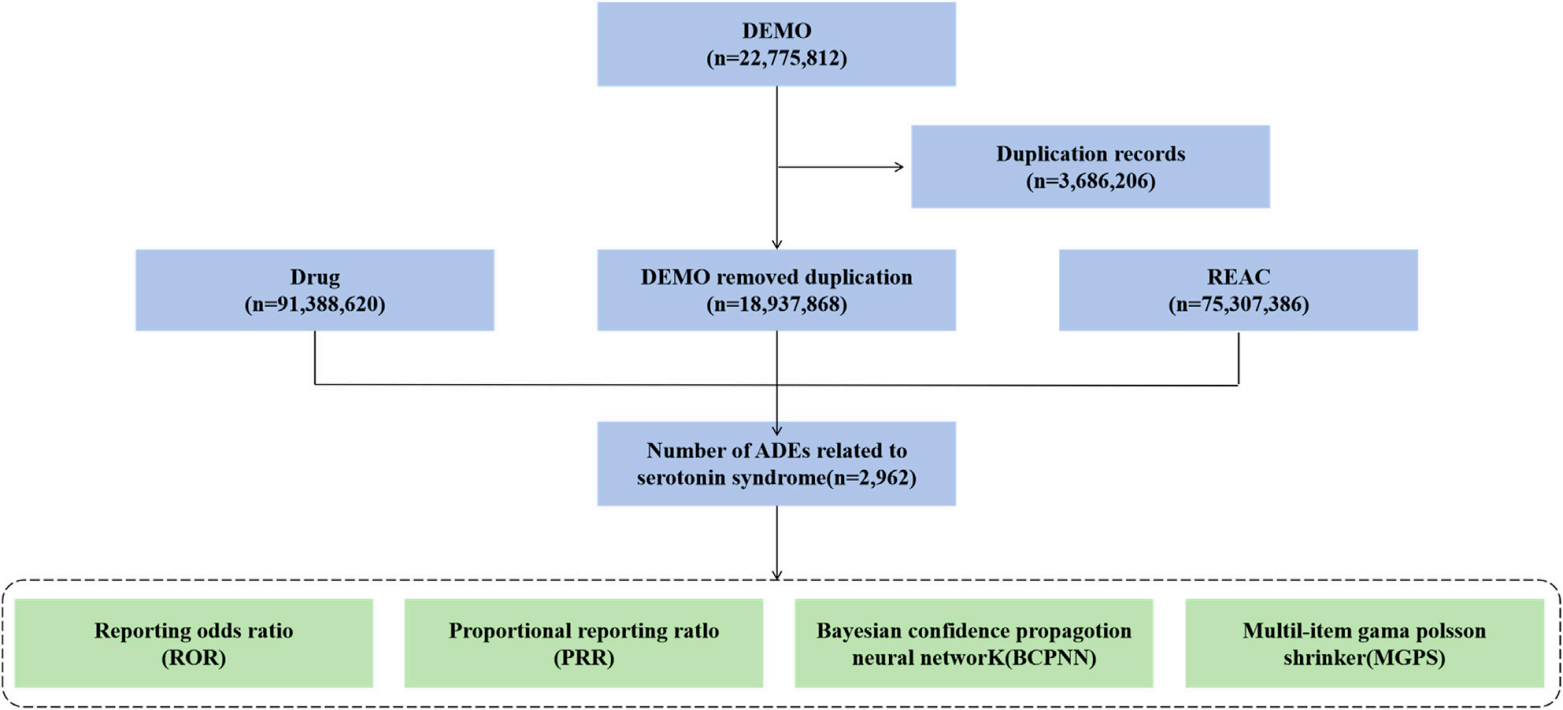
Flow chart of the study for drug-associated serotonin syndrome data in the FAERS database. Abbreviations: DEMO, patient demographic and administrative information; DRUG, drug information; REAC, adverse events; ADEs, Adverse Drug Events.

This retrospective, real-world pharmacovigilance study employed data from the FAERS database. Managed by the FDA, FAERS is a comprehensive database that collects spontaneous reports of adverse events and medication errors from healthcare professionals, consumers, and manufacturers. The database consists of seven distinct datasets, which include: patient demographic and administrative information (DEMO), drug information (DRUG), adverse events (REAC), patient outcomes (OUTC), report sources (RPSR), therapy start and end dates for the reported drugs (THER), and indications for drug administration (INDI). This study analyzed data from the first quarter of 2004 through the first quarter of 2025, encompassing a 21-year period of postmarketing safety surveillance. Patient age was defined as ≥65 years. Adverse events were identified by the preferred term (PT) “Serotonin syndrome” as defined in the Medical Dictionary for Regulatory Activities (MedDRA, version 26.1). From these, primary suspected drugs associated with serotonin syndrome were extracted. Generic names and trade names were cross-referenced via the DrugBank database (www.drugbank.com). To categorize drugs into therapeutic categories and facilitate the analysis of their associations with adverse events, the detected signal drugs were classified according to the Anatomical Therapeutic Chemical (ATC) classification system. To ensure methodological transparency and reproducibility, this study adhered to the READUS-PV guideline (Fusaroli et al., 2024a; Fusaroli et al., 2024b). The flow of data processing is detailed in Figure 1.

### 2.2 Disproportionality analysis

To identify signal drugs associated with serotonin syndrome using the adverse event reporting database, four algorithms were employed to detect drug-event combinations with signals exceeding expected frequencies: Reported Odds Ratio (ROR), Proportional Reporting Ratio (PRR), Bayesian Confidence Propagation Neural Network (BCPNN), and Multi-item Gamma Poisson Shrinker (MGPS). Detailed formulas and criteria for these algorithms are presented in Supplementary Table S1. A drug was deemed positive only if all four methods yielded positive results. Moreover, volcano plots were generated to visually compare the differences in serotonin syndrome signals among different drugs in elderly patients. These volcano plots were created using the ROR along with the adjusted P-value obtained from Fisher’s exact test followed by Bonferroni correction, which enables a clear visualization of the differences in serotonin syndrome signals of different drugs in elderly patients. Additionally, we ascertained whether these drugs carried a serotonin syndrome warning by consulting the Summary of Product Characteristics (SPCs) issued by the FDA.

### 2.3 Sensitivity analysis

The qualifications of reporters may introduce potential biases in our study. To enhance data quality and minimize erroneous reports, cases reported by non-professional reporters (e.g., consumers) were excluded. Disproportionality analysis was specifically conducted on serotonin syndrome cases reported by healthcare professionals (e.g., physicians, and pharmacists).

### 2.4 Time-to-onset analysis

Time-to-onset (TTO) was defined as the interval between EVENT_DT (the date of adverse event onset) and START_DT (the date of drug initiation). Given the markedly non-normal distribution of drug-associated TTO, quartiles were employed for descriptive statistics, and median TTO values were compared across different drug categories. Additionally, cumulative distribution curves were constructed to characterize the time-to-onset characteristics of drug-associated serotonin syndrome across genders, age groups, and drug categories.

### 2.5 Statistical analysis

For disproportionality analysis, a drug was deemed a signal drug for further investigation if it met the following criteria: lower 95% confidence interval (CI) of ROR (ROR025) > 1, PRR ≥2, Chi-square (χ^2^) ≥ 4, lower 95% confidence interval (CI) of IC (IC025) > 0, and the number of reported serotonin syndrome cases (a) ≥ 3. In further stratified analyses by gender, age group, and drug category, the Mann-Whitney test and Kruskal-Wallis test were employed to compare differences in median time-to-onset values. Prior to time-to-onset analysis, erroneous or missing data were excluded from the dataset, and only drugs with at least two valid data points were included in the analysis. All statistical analyses were conducted using R (version 4.5.0) and RStudio software.

## 3 Results

### 3.1 Descriptive analysis

Among the 2,962 cases documented in the FAERS database, 2,494 were specifically reported by healthcare professionals. A total of 236 drugs were identified for the analysis of drug-associated serotonin syndrome. Among the reported cases, female patients accounted for a larger proportion (61.5%) compared to male patients (37.6%). The majority of patients fell within the age range of 65–74 years. The most frequent adverse outcome was hospitalization, followed by other serious outcomes and life-threatening events. The majority of reports originated from the United States (34.4%), followed by Spain and the United Kingdom. Since 2008, reports of drug-associated adverse reactions linked to serotonin syndrome have exhibited a fluctuating upward trend, with a peak observed in 2024. The demographic and clinical characteristics of the included patients are summarized in Table 1.

**TABLE 1:** Clinical characteristics of patients with drug-associated serotonin syndrome in the FAERS databases.

Characteristics	Number (%)
Number of patients	2,962
Gender	
Female	1823 (61.5%)
Male	1,114 (37.6%)
Missing	25 (0.8%)
AGE(year)	
65–74	1798 (60.7%)
75–84	868 (29.3%)
85–94	254 (8.6%)
≥95	42 (1.4%)
Reported countries (Top 10)	
United States	1,020 (34.4%)
Spain	268 (9.0%)
United Kingdom	238 (8.0%)
France	148 (5.0%)
Japan	133 (4.5%)
Canada	100 (3.4%)
Italy	77 (2.6%)
Germany	60 (2.0%)
Netherlands	59 (2.0%)
Portugal	51 (1.7%)
Outcome	
Death	219 (7.4%)
Disability	21 (0.7%)
Hospitalization	1,375 (46.4%)
Life-Threatening	544 (18.4%)
Other	803 (27.1%)
Reporter type	
Healthcare professionals	2,494 (84.2%)
Non-healthcare professionals	293 (9.9%)
Missing	175 (5.9%)
Received year	
2004	62 (2.1%)
2005	52 (1.8%)
2006	86 (2.9%)
2007	63 (2.1%)
2008	61 (2.1%)
2009	74 (2.5%)
2010	90 (3.0%)
2011	137 (4.6%)
2012	124 (4.2%)
2013	149 (5.0%)
2014	113 (3.8%)
2015	167 (5.6%)
2016	141 (4.8%)
2017	163 (5.5%)
2018	202 (6.8%)
2019	224 (7.6%)
2020	195 (6.6%)
2021	129 (4.4%)
2022	154 (5.2%)
2023	205 (6.9%)
2024	328 (11.1%)
2025Q1	43 (1.5%)

### 3.2 Disproportionality analysis

**FIGURE 2:**
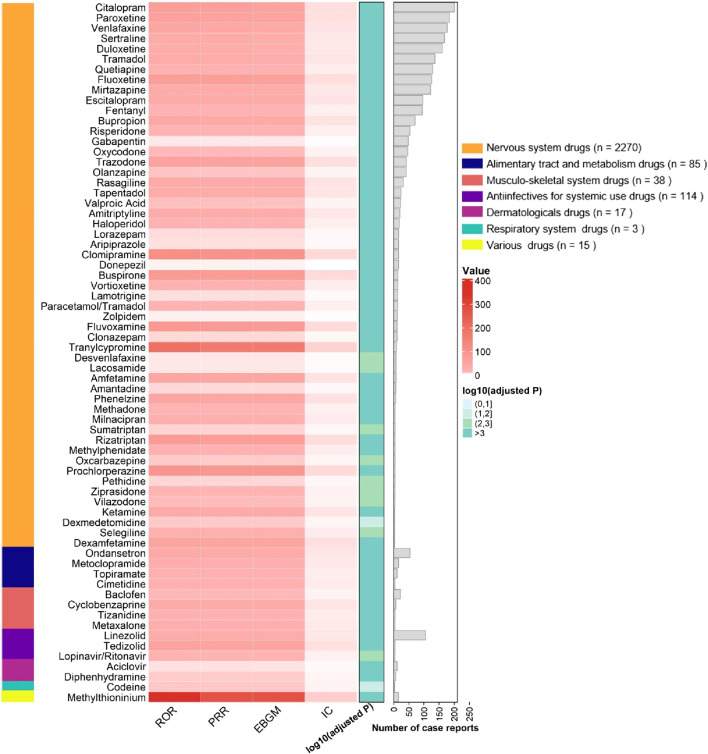
Signaling Drugs Associated with Serotonin Syndrome in Elderly Patients from the FAERS Database. Healthcare professional reports (n = 2,494) are included in the FAERS total (n = 2,962).

**FIGURE 3:**
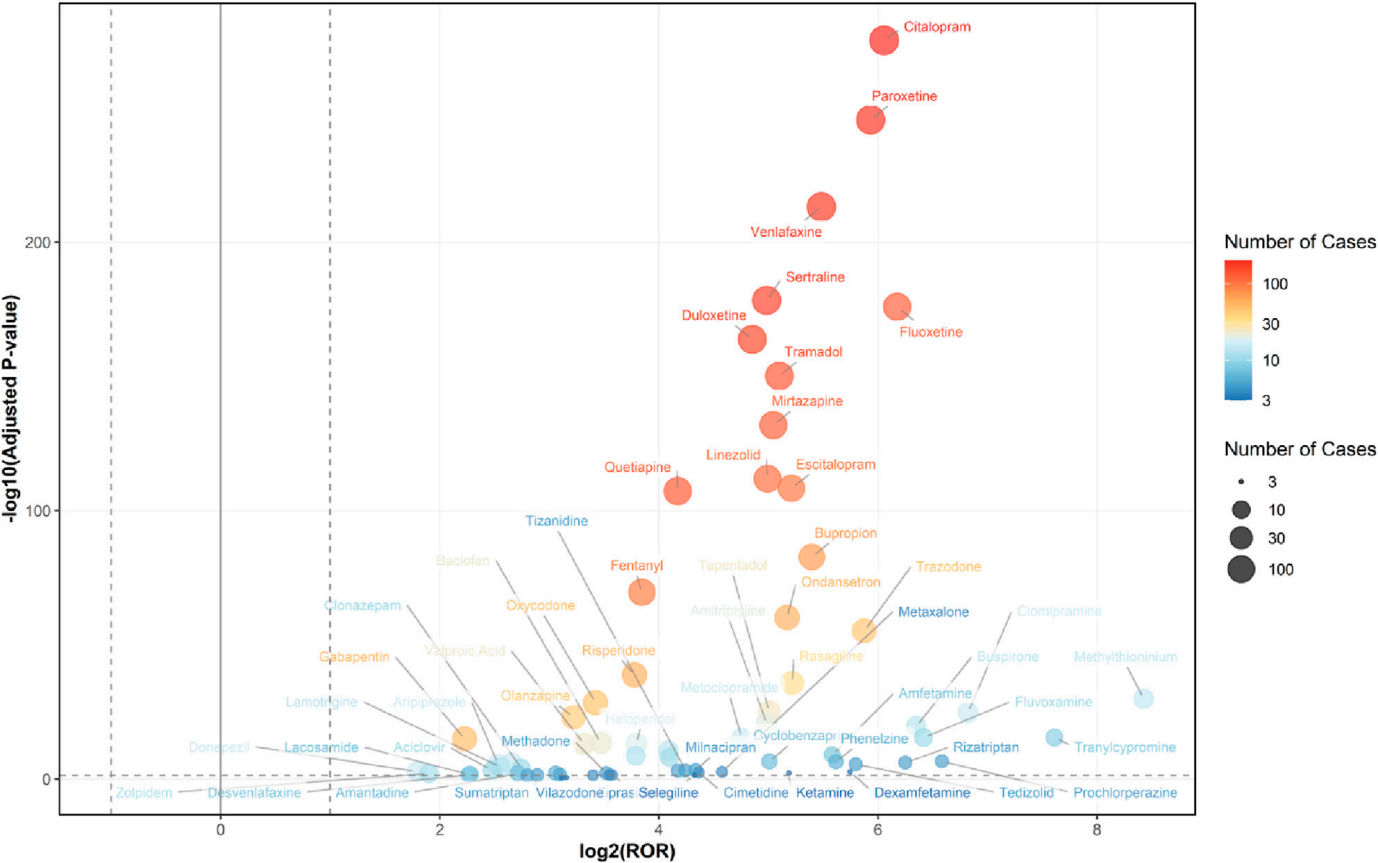
Volcano Plot for Comparing Serotonin Syndrome Signals Among Different Drugs in Elderly Patients. The x-axis represents the logarithmic value of the ROR, while the y-axis denotes the negative base-10 logarithm of the adjusted P-value. This adjusted P-value was derived using Fisher’s exact test followed by Bonferroni correction. The color intensity of each data point indicates the number of reports, where warmer colors (e.g., reddish hues) correspond to a higher number of reports. Drugs positioned in the upper right quadrant exhibit both greater signal intensity and statistically significant differences. Healthcare professional reports (n = 2,494) are included in the FAERS total (n = 2,962).

Among the 236 drugs analyzed, signals were detected for 68 agents, with their classification based on ATC codes provided in Supplementary Table S2. Among the drugs with positive signals, the most frequently reported category associated with serotonin syndrome was nervous system drugs, accounting for 2,270 reports (76.7%). This was followed by antiinfectives for systemic use (114 reports, 3.8%), alimentary tract and metabolism drugs (85 reports, 2.9%), musculo-skeletal system drugs (38 reports, 1.3%), dermatologicals (17 reports, 0.6%), various drugs (15 reports, 0.5%), and respiratory system drugs (3 reports, 0.1%). Details regarding this are provided in Figures 2, 3.

### 3.3 Sensitivity analysis

**FIGURE 4:**
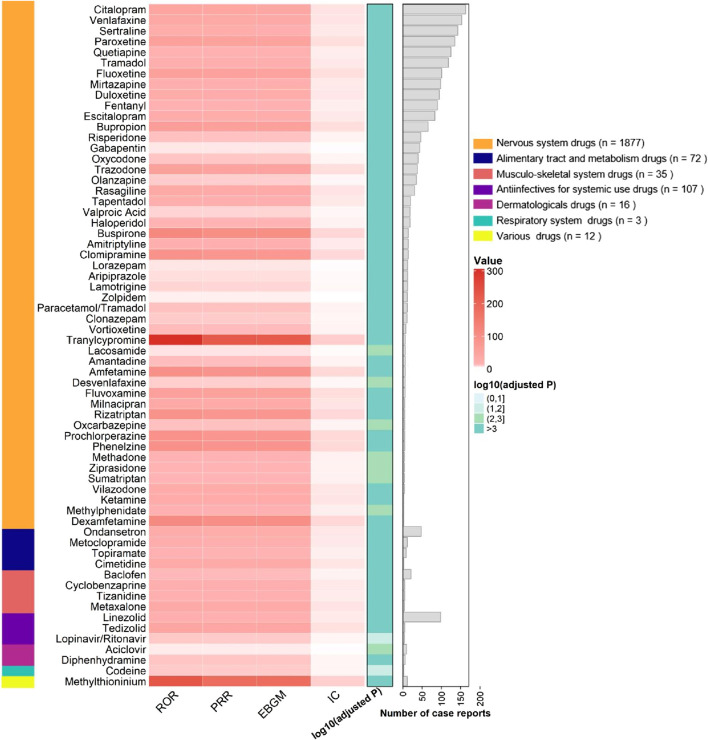
Signaling Drugs Associated with Serotonin Syndrome from FAERS Database, based on reports from healthcare professionals. Healthcare professional reports (n = 2,494) are included in the FAERS total (n = 2,962).

**FIGURE 5:**
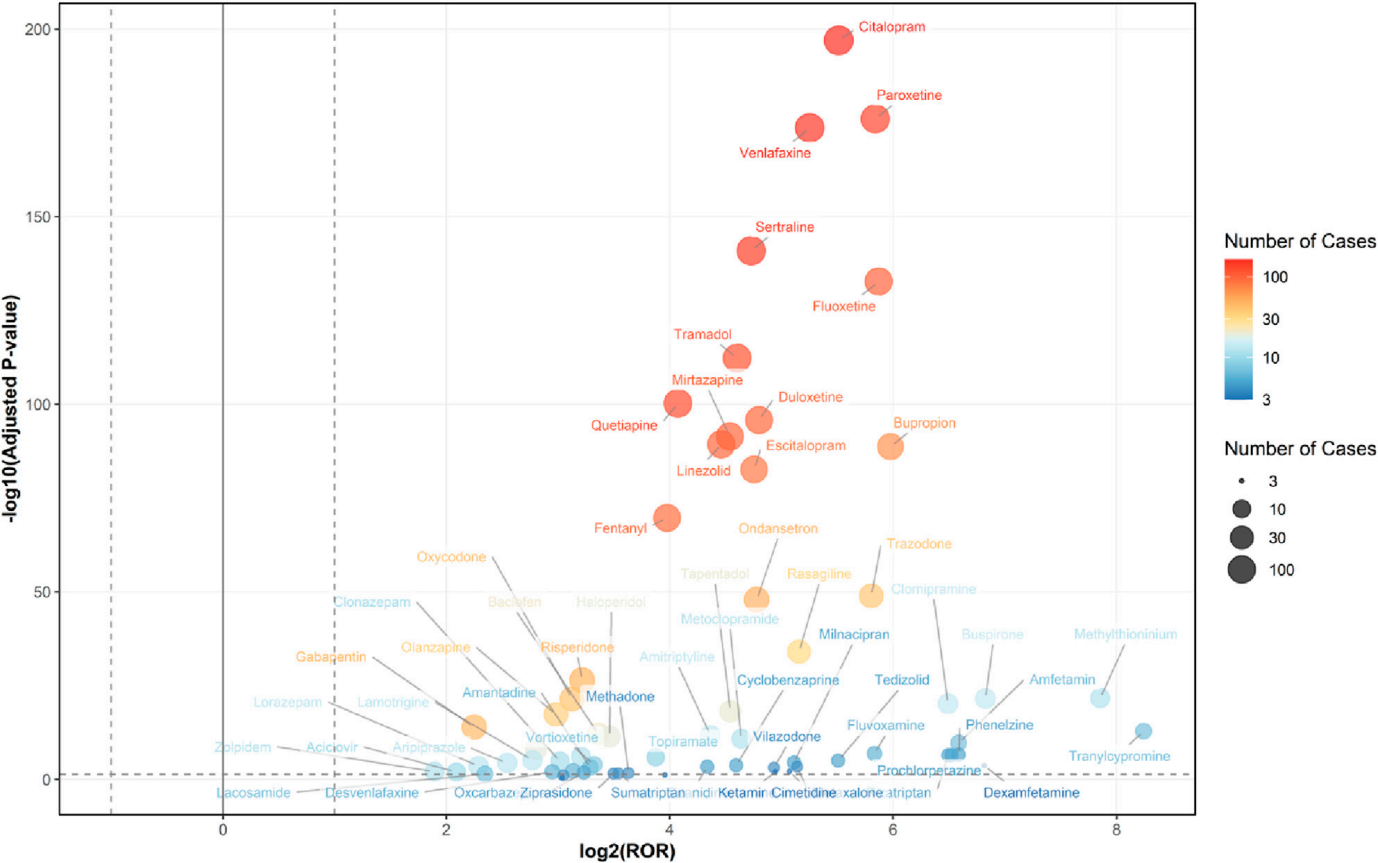
Volcano Plot for Comparing Serotonin Syndrome Signals Among Different Drugs in Elderly Patients, based on reports from healthcare professionals. The x-axis represents the logarithmic value of the ROR, while the y-axis denotes the negative base-10 logarithm of the adjusted P-value. This adjusted P-value was derived using Fisher’s exact test followed by Bonferroni correction. The color intensity of each data point indicates the number of reports, where warmer colors (e.g., reddish hues) correspond to a higher number of reports. Drugs positioned in the upper right quadrant exhibit both greater signal intensity and statistically significant differences. Healthcare professional reports (n = 2,494) are included in the FAERS total (n = 2,962).

From the 2,494 cases reported by healthcare professionals, a total of 205 drugs were identified for analyzing drug-related serotonin syndrome. Among these 205 analyzed drugs, signals were detected for 64 agents (Figures 4, 5), with their classification based on ATC codes provided in Supplementary Table S3. The analytical findings demonstrated that the drugs with disproportionality signals reported by healthcare professionals in the FAERS database were largely consistent with those identified in the overall reports from all reporters.

### 3.4 Time-to-onset and cumulative distribution curve analysis

**FIGURE 6:**
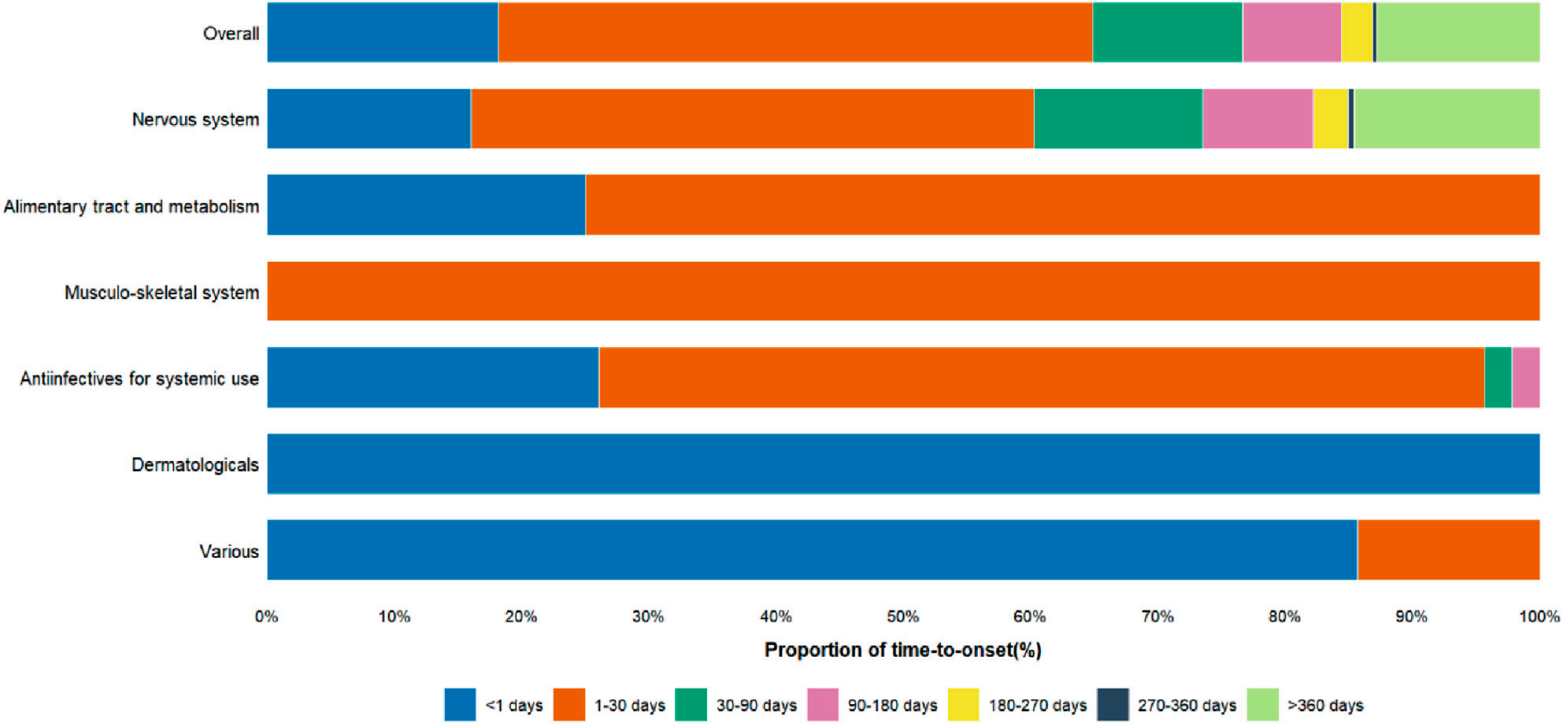
Proportion of Time-to-onset of Serotonin Syndrome in Elderly Patients. The x-axis represents the proportion of time-to-onset, categorized into various time intervals, while the y-axis shows the therapeutic drug classes associated with Serotonin Syndrome.

The proportions of cases with a TTO of <1 day were 18.16% in the overall cohort, 15.98% among nervous system drugs, 25.00% among alimentary tract and metabolism drugs, 26.09% among antiinfectives for systemic use, 100% among dermatologicals, and 85.71% among various drugs (Figure 6).

**FIGURE 7:**
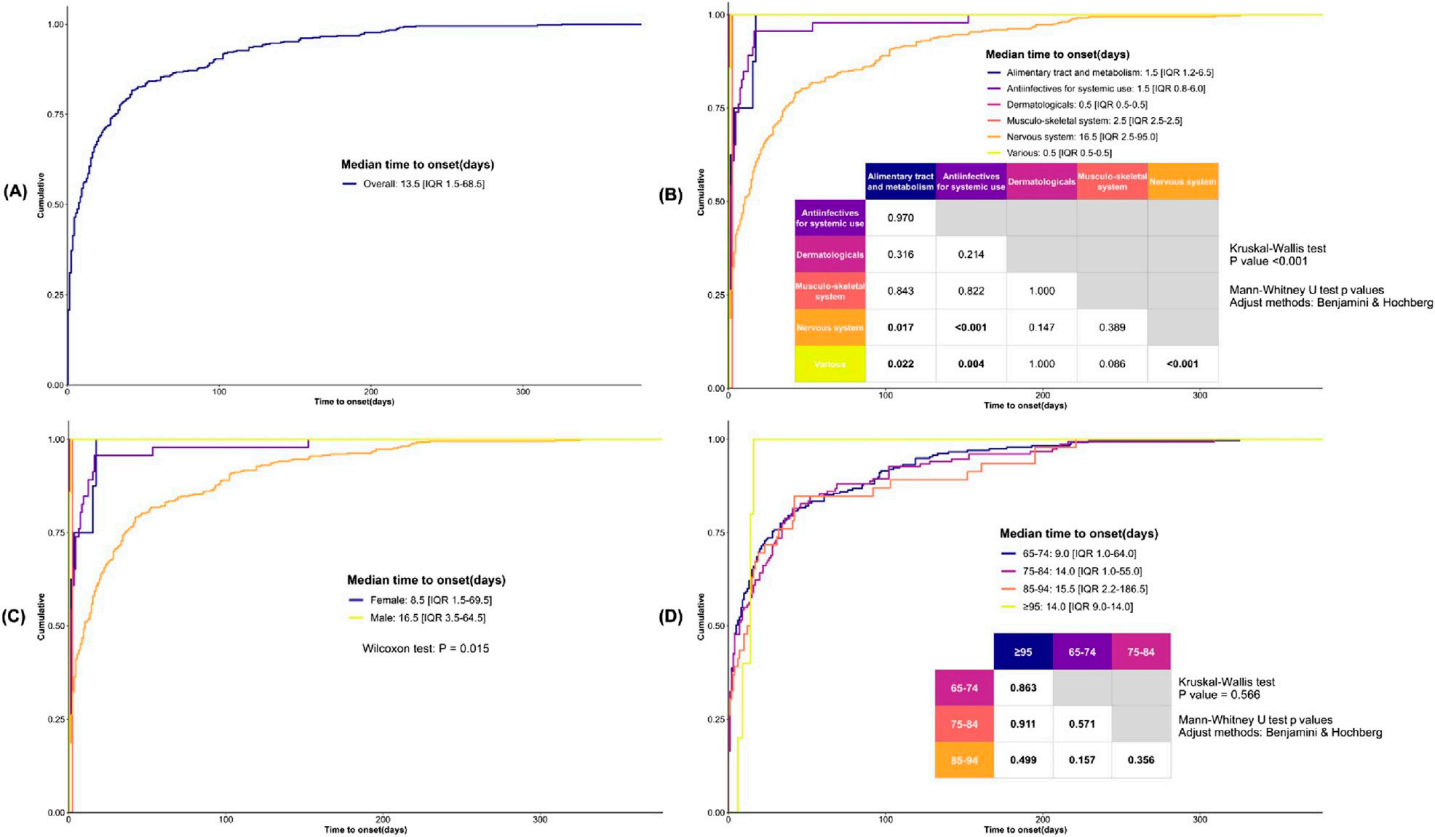
Analysis of Time-to-Onset of Serotonin Syndrome in Elderly Patients. **(A)** The cumulative distribution curve illustrates the overall time-to-onset of serotonin syndrome. **(B)** The cumulative distribution curve illustrates the time-to-onset of serotonin syndrome across various drug categories. **(C)** The cumulative distribution curve illustrates the time-to-onset of serotonin syndrome across patients of different genders. **(D)** The cumulative distribution curve illustrates the time-to-onset of serotonin syndrome across patients from different age groups. Statistical analyses were performed using the Mann-Whitney U test, with pairwise comparisons conducted via the Kruskal-Wallis test and adjustments applied using the Benjamini & Hochberg method.

The overall TTO of drug-associated serotonin syndrome is 13.5 days [interquartile range (IQR): 1.5–68.5 days] (Figure 7A). To further explore the potential factors influencing time-to-onset, we performed additional analyses stratified by drug categories, gender, and age. Due to limited data availability for respiratory system drugs (only 3 data points, with TTO data missing), further stratification was not feasible, and thus these drugs were excluded from the analysis. Regarding drug categories (Figure 7B), the TTO for nervous system drugs was 16.5 days [IQR: 2.5–95.0 days], for antiinfectives for systemic use was 1.5 days [IQR: 0.8–6.0 days], for alimentary tract and metabolism drugs was 1.5 days [IQR: 1.2–6.5 days], for musculo-skeletal system drugs was 2.5 days [IQR: 2.5–2.5 days], for dermatologicals was 0.5 days [IQR: 0.5–0.5 days], and for various drugs was 0.5 days [IQR: 0.5–0.5 days]. Cumulative distribution curve analysis revealed that the median time-to-onset of nervous system drugs differed significantly from that of antiinfectives for systemic use, alimentary tract and metabolism drugs, and various drugs. In terms of gender, serotonin syndrome onset occurred earlier in elderly females (8.5 days [IQR: 1.5–69.5 days]) and later in elderly males (16.5 days [IQR: 3.5–64.5 days]) (Figure 7C). Additionally, age exerted no significant influence on the time-to-onset of drug-related serotonin syndrome (Figure 7D).

## 4 Discussion

We conducted a retrospective analysis of adverse event reports involving elderly patients recorded in the FAERS, the largest global repository of spontaneous adverse event reports. Drug-associated serotonin syndrome in geriatric patients requires heightened vigilance, as it is associated with longer hospital stays and may be fatal in severe cases. It has been reported that drug-related adverse events account for up to 30% of hospital admissions among elderly patients (Chan et al., 2001; Salvi et al., 2012). With the global aging of the population, increased attention should be directed toward the risk of drug-associated serotonin syndrome in elderly patients.

The association of adverse events related to serotonin syndrome was assessed by ROR, PRR, BCPNN, and MGPS analyses. Sixty-eight drugs met the predefined signal criteria and were flagged as potential inducers of serotonin syndrome in elderly patients. Most positive signals were observed in drugs acting on the nervous system drugs, alimentary tract and metabolism drugs, musculo-skeletal system drugs, antiinfectives for systemic use, dermatologicals, respiratory system drug. For all drugs that produced positive signals, the ROR was greater than 1.0, reflecting an increased reporting frequency of serotonin syndrome relative to the background reporting rate.

These results are consistent with previous literature, including systematic reviews, case reports, and FDA pharmacovigilance studies, in that SSRIs, serotonin norepinephrine reuptake inhibitors (SNRIs), MAOIs, and other classic serotonergic agents continue to account for the majority of reported serotonin syndrome cases (Boyer and Shannon, 2005; Finberg and Rabey, 2016; Bartlett, 2017; Spadaro et al., 2022; Mikkelsen et al., 2023; Elli et al., 2024). Of the drug classes evaluated, SSRIs were most frequently implicated as causative agents of serotonin syndrome (Gummin et al., 2024).

Notably, several medications that were not previously recognized as serotonin syndrome inducers exhibited signals for serotonin syndrome in older patients. Of particular concern were atypical antipsychotics (quetiapine, risperidone, olanzapine, aripiprazole), opioid analgesics (oxycodone, methadone), anticonvulsants (lamotrigine, lacosamide), and gabapentinoids (gabapentin). The associations persisted in a sensitivity analysis restricted to reports submitted by healthcare professionals. Evidence indicates that concurrent use of serotonergic agents elevates the risk of serotonin syndrome, and the highest frequency and severity of cases are typically associated with co-administration of SSRIs and MAOIs(Cooper et al., 2023; Isbister et al., 2003; Gillman, 2005). Polypharmacy is prevalent in older adults, and drug interactions among concomitant medications may increase the risk of serotonin toxicity.

In older patients, the median TTO of drug-related serotonin syndrome was 13.5 days [IQR:1.5–68.5 days], which is substantially longer than the rapid-onset pattern typically emphasized in earlier case series (Mason et al., 2000); however, this finding is concordant with other reports that document considerable variability in the syndrome’s incubation period (Werneke et al., 2016). In a stratified analysis by therapeutic class, drugs acting on the nervous system had a median TTO of 16.5 days [IQR: 2.5–95.0 days], whereas antiinfectives for systemic use and alimentary tract and metabolism drugs exhibited markedly shorter median TTOs of 1.5 days. Overall, 18.16% of cases had a TTO of less than 24 h. The proportion was 16.0% for cases associated with drugs acting on the nervous system and ranged from 25% to 100% across other therapeutic classes. These data indicate that drug-related serotonin syndrome in older patients may present either acutely (within hours to 24 h) or subacutely (days to weeks), with agents acting on the nervous system more often associated with delayed onset. In clinical practice, clinicians should remain vigilant after drug initiation or dose adjustment for both immediate-onset serotonin syndrome and presentations that emerge over the following weeks; central nervous system agents in particular require close, continued monitoring after initiation or dose escalation.

This study has several limitations. First, adverse event reports in the FAERS database are voluntarily submitted, which may give rise to underreporting, misreporting, and reporting bias. Second, methodological limitations preclude us from establishing a causal relationship between drugs and the observed positive signals or reported cases. Finally, the paucity of key data in the FAERS database may introduce potential biases into our research. For instance, the number of reports with clearly documented TTO was small, which limits the precision of subgroup estimates for the incubation period.

## 5 Conclusion

Analysis of the FAERS shows a substantial burden of drug-induced serotonin syndrome in elderly patients; because medications are a major and potentially preventable risk factor, the risk of serotonin syndrome should be explicitly considered when optimizing pharmacotherapeutic regimens.

## Data Availability

Publicly available datasets were analyzed in this study. This data can be found here: https://fis.fda.gov/extensions/FPD-QDE-FAERS/FPD-QDE-FAERS.html.
